# Chromosome-scale genome assembly of *Sauvagesia rhodoleuca* (Ochnaceae) provides insights into its genome evolution and demographic history

**DOI:** 10.1093/dnares/dsaf022

**Published:** 2025-09-02

**Authors:** Tian-Wen Xiao, Xin-Feng Wang, Zheng-Feng Wang, Hai-Fei Yan

**Affiliations:** College of Biology and Food Engineering, Guangdong University of Education, Guangzhou 510303, China; Key Laboratory of National Forestry and Grassland Administration on Plant Conservation and Utilization in Southern China, South China Botanical Garden, Chinese Academy of Sciences, Guangzhou 510650, China; South China National Botanical Garden, Guangzhou 510650, China; Key Laboratory of National Forestry and Grassland Administration on Plant Conservation and Utilization in Southern China, South China Botanical Garden, Chinese Academy of Sciences, Guangzhou 510650, China; South China National Botanical Garden, Guangzhou 510650, China; Guangdong Provincial Key Laboratory of Applied Botany, South China Botanical Garden, Chinese Academy of Sciences, Guangzhou 510650, China; State Key Laboratory of Plant Diversity and Specialty Crops, South China Botanical Garden, Chinese Academy of Sciences, Guangzhou 510650, China; Key Laboratory of National Forestry and Grassland Administration on Plant Conservation and Utilization in Southern China, South China Botanical Garden, Chinese Academy of Sciences, Guangzhou 510650, China; South China National Botanical Garden, Guangzhou 510650, China; Guangdong Provincial Key Laboratory of Applied Botany, South China Botanical Garden, Chinese Academy of Sciences, Guangzhou 510650, China; Key Laboratory of Vegetation Restoration and Management of Degraded Ecosystems, South China Botanical Garden, Chinese Academy of Sciences, Guangzhou 510650, China; Key Laboratory of National Forestry and Grassland Administration on Plant Conservation and Utilization in Southern China, South China Botanical Garden, Chinese Academy of Sciences, Guangzhou 510650, China; South China National Botanical Garden, Guangzhou 510650, China; Guangdong Provincial Key Laboratory of Applied Botany, South China Botanical Garden, Chinese Academy of Sciences, Guangzhou 510650, China; State Key Laboratory of Plant Diversity and Specialty Crops, South China Botanical Garden, Chinese Academy of Sciences, Guangzhou 510650, China

**Keywords:** *Sauvagesia rhodoleuca*, genome assembly, whole-genome duplication, effective population size, evolution

## Abstract

*Sauvagesia rhodoleuca* is an endangered species endemic to southern China. Due to human activities, only 6 fragmented populations remain in Guangdong and Guangxi. Despite considerable conservation efforts, its demographic history and evolution remain poorly understood, particularly from a genomic perspective. To address this, we assembled a chromosome-scale genome of *S. rhodoleuca* using Nanopore long-read sequencing, DNA short-read sequencing, RNA-seq, and Hi-C data. A total of 290.37 Mb of assembled sequences, accounting for 99.76% of the genome, were successfully anchored to 19 pseudo-chromosomes, achieving a BUSCO completeness of 98.40% and a long terminal repeat assembly index of 17.28. Genome annotation identified 26,758 protein-coding genes and 369 tRNA genes. Demographic analysis revealed a sharp decline in the effective population size of *S. rhodoleuca* beginning approximately 1 million years ago. Whole-genome duplication (WGD) analysis revealed that *S. rhodoleuca* experienced a whole-genome triplication (WGT) followed by a more recent WGD after diverging from the Rhizophoraceae. Genes retained from WGT and WGD events played key roles in the development and survival of *S. rhodoleuca*, as indicated by Gene Ontology analysis. The high-quality genome of *S. rhodoleuca* provides insights into its genomic characteristics and evolutionary history, offering a valuable resource for conservation and genetic management.

## 1. Introduction

Ochnaceae (Malpighiales) is a pantropical family comprising around 27 genera and 500 species.^1^ Within this family, *Sauvagesia* L. is notable for its broad distribution across tropical Asia, Africa, and America.^1,2^ This genus consists of approximately 35 species, primarily shrubs, with occasional small trees or herbs.^3^*Sauvagesia rhodoleuca* (Diels) M. C. E. Amaral, a shrub endemic to China, is restricted to Guangdong and Guangxi.^4,5^ The roots and stems of *S. rhodoleuca* have medicinal properties and have traditionally been used for therapeutic purposes. However, due to overexploitation and deforestation, *S. rhodoleuca* is on the brink of extinction and has already vanished from parts of its historical range.^4^ Consequently, it has been designated a Class II protected wild plant in China, emphasizing the urgency of conservation efforts. However, there is still a lack of in-depth knowledge about this species, especially from the genetic aspects.

First, the historical changes in the effective population size of *S. rhodoleuca* remain unclear. The pairwise sequentially Markovian coalescent approach is widely used to estimate changes in effective population size over time.^6^ Such analyses enhance our understanding of the factors driving population size fluctuations, including biotic interactions and extrinsic environmental changes. For example, population histories of temperate walnut species and sibling grouse species have been shown to be influenced by the dramatic climatic oscillations of the Quaternary.^7,8^ Similarly, the current small population size of *S. rhodoleuca* may reflect a long-term decline, as observed in co-distributed species such as *Euryodendron excelsum* Hung T. Chang (Pentaphylacaceae), for which historical climate change has been suggested as a major contributing factor.^9^

Second, clarification is needed regarding the extent of whole-genome duplication (WGD) events in this species. WGD, also known as polyploidization, is common during angiosperm evolution.^10^ Many plant lineages experienced WGD near the Cretaceous–Paleogene boundary, potentially providing an adaptive advantage in stressful conditions and periods of environmental upheaval.^11–14^ Malpighiales, an order with approximately 16,000 species, exhibits remarkable morphological and ecological diversity.^15^ Many species within this order are economically significant crops, such as cassava, passion fruit, and rubber. As a result, several studies have explored WGD in this highly diverse lineage to uncover the molecular mechanisms underlying various traits.^16–19^ For example, transcriptomic data indicate that *Ochna* L. (Ochnaceae) underwent 2 rounds of WGD after diverging from the *Clusia*-*Mammea* clade.^19^ However, high-quality genome assemblies can provide a more comprehensive and reliable framework for inferring WGD events in Ochnaceae compared to transcriptomic data. Therefore, establishing a high-quality reference genome for *S. rhodoleuca* is critical for advancing evolutionary and genomic studies in this family.

Third, the phylogenetic position of Ochnaceae within Malpighiales remains to be elucidated. Phylogenies are essential for addressing diverse biological questions, including species relationships, population structure, gene family evolution, and the origin and dispersal of species.^20^ Given the ecological and economic significance of Malpighiales, a well-resolved phylogeny is crucial for evolutionary and genomic studies, as well as crop improvement.^21^ Previous studies examined phylogenetic relationships within Malpighiales using either a few molecular markers^15^ or hundreds of nuclear genes^22^; however, phylogenetic positions of many families, including Ochnaceae, remain unresolved. This uncertainty may stem from the rapid radiation of Malpighiales in the mid-Cretaceous^21,23^ or the limited genomic data available in previous studies. With advances in third-generation sequencing, high-quality genomes have increasingly been employed to resolve challenging phylogenetic nodes across the tree of life.^24–26^ Accordingly, a high-quality genome of *S. rhodoleuca* would contribute to resolving phylogenetic relationships within Malpighiales.

However, the lack of such a reference genome has hindered efforts to investigate these questions in depth. To address this, we assembled a chromosome-scale genome of *S. rhodoleuca*—the first reported for the Ochnaceae family—using Oxford Nanopore Technology (ONT) long reads, high-throughput chromosome conformation capture (Hi-C) reads, DNA short reads, and RNA-seq reads. We conducted phylogenetic analyses, reconstructed historical effective population size dynamics, and examined WGD events in *S. rhodoleuca*. This genome provides a valuable resource for the conservation of *S. rhodoleuca* and for advancing evolutionary studies of Malpighiales.

## 2. Materials and methods

### 2.1. Plant materials

Genome sequencing materials of *S. rhodoleuca* were collected from Heishiding Provincial Nature Reserve in Fengkai city (111°53’60’ E, 23°27’50’ N, altitude 199 m), Guangdong province, China. Voucher specimen (ID: gexj230011) has been deposited in the herbarium of the South China Botanical Garden, Chinese Academy of Sciences (IBSC). Materials were immediately frozen in liquid nitrogen after collection and subsequently stored in a refrigerator at −80 °C for DNA and RNA extraction.

### 2.2. Oxford Nanopore Technology sequencing

Total DNA was extracted using Grandomics Genomic DNA Kit (GrandOmics Biosciences, Wuhan, China) following the manufactural protocol. DNA quality was assessed by a 0.75% gel electrophoresis experiment, and quantified using a NanoDrop One UV–Vis spectrophotometer (Thermo Fisher Scientific, Waltham, MA, USA). Nanopore library was prepared with the LSK109 Ligation Sequencing Kit and then sequenced on a Nanopore PromethION sequencer (Oxford Nanopore Technologies, Oxford, UK) at Grand Omics Co., Ltd (Wuhan, China).

### 2.3. Short-read sequencing

For DNA short-read sequencing, a paired-end library (2 × 150 bp) was constructed using the TruSeq Nano DNA HT Sample Preparation Kit (Illumina). DNA sequencing was then performed on the MGI DNBSEQ-T7 platform (hereafter referred to as DNBSEQ short reads) at Grand Omics Co., Ltd.

To facilitate genome annotation, total RNA was extracted from leaves, twigs, and fruits using the TRNzol Universal RNA Extraction Kit (Tiangen, Beijing, China). A paired-end RNA library (2 × 150 bp) was constructed using TruSeq RNA Library Preparation Kit (Illumina), and sequenced on the MGI DNBSEQ-T7 platform (BGI, Shenzhen, China) at Grand Omics Co., Ltd.

### 2.4. Hi-C sequencing

Young fresh leaves were fixed in 2% formaldehyde, and the cross-linked chromatin was digested with *DpnII* (New England Biolabs, USA). The digested DNA ends were labeled with biotin-14-dCTP and ligated using T4 DNA polymerase (New England Biolabs). The ligated products were enriched, sheared into 300–600 bp fragments, blunt-end repaired, A-tailed, and purified using biotin-streptavidin-mediated pull-down. A 150 bp paired-end Hi-C library was then constructed and sequenced on the MGI DNBSEQ-T7 platform at Grand Omics Co., Ltd.

### 2.5. Quality control

For Nanopore reads, adapters were removed using Porechop v0.2.4.^27^ For short-read data and Hi-C reads, adapters and low-quality reads were trimmed using fastp v0.23.3^28^ with default parameters.

### 2.6. *De novo* genome assembly and quality assessment

Before genome assembly, DNBSEQ short reads were used to estimate genome size using Jellyfish v2.3.0^29^ and GenomeScope v1.0^30^ with a k-mer length of 21. Organellar sequences in Nanopore reads were removed by aligning the reads to the plastid and mitochondrial genomes of *S. rhodoleuca* and *Salix pseudolasiogyne* H. Lév. (GenBank accession numbers MW772237 and PQ873111), and subsequently discarding the mapped reads. Filtered Nanopore reads were used for genome assembly via NextDenovo v2.5.1.^31^ Purge Haplotigs v1.1.2^32^ was used to remove haplotypic duplications based on the coverage information from Nanopore reads. The genome assembly was subsequently polished using Nanopore reads for 2 rounds via Racon v1.5.0^33^ and DNBSEQ short reads for 2 rounds via Polypolish v0.5.0.^34^ The polished genome was scaffolded with Hi-C reads using HapHiC v1.0.6^35^ and manually adjusted in Juicebox v1.11.08.^36^ The genome assembly was gap-filled based on Nanopore reads using TGS-GapCloser v1.1.1,^37^ which was further polished as described above.

The quality of genome assembly was assessed using the Benchmarking Universal Single-Copy Orthologues (BUSCO v5.3.2)^38^ with the database embryophyta_odb10.2020-09-10. To evaluate genome completeness, DNBSEQ short reads were mapped to genome assembly using BWA-MEM v0.7.17-r1188,^39^ and the percentage of mapped reads was summarized with the function ‘stats’ in BamTools v2.5.1.^40^ The tool Clipping Reveals Assembly Quality (CRAQ) v1.0.9^41^ was used to identify regional and structural assembly errors by mapping DNBSEQ short reads and Nanopore reads to the genome assembly.

### 2.7. Genome annotation

Repetitive elements within the genome of *S*. *rhodoleuca* were identified using the Extensive *de novo* TE Annotator (EDTA) v2.1.0.^42^ The long terminal repeat (LTR) assembly index (LAI) was calculated based on the outputs of EDTA using the LAI program implemented in the LTR_retriever v2.9.0.^43^ LTR insertion time was estimated using LTR_retriever, which calculates sequence divergence (K) between LTR pairs based on the Jukes–Cantor (1969) model^44^, and then estimates insertion time using the formula *T* = *K*/(2*r*), where *r* is the nucleotide substitution rate, assumed to be 1.3 × 10^−8^ substitutions per site per year.^43^ Repetitive elements in the genome assembly were soft-masked using RepeatMasker v4.1.2^45^ with a custom repeat library generated by EDTA. The soft-masked genome assembly was used for gene prediction via the funannotate v1.8.15 pipeline^46^ with a combination of *de novo*, RNA-seq-based, and homology-based annotation approaches following Xiao et al.,^47^ who combined *ab initio* prediction, transcriptome evidence, and protein homology from related species, and refined gene models using Program to Assemble Spliced Alignments (PASA) and evidence weighting strategies to optimize gene structure annotation. In the funannotate pipeline, Augustus v3.5.0^48^ and GeneMark-ES v4.7.1^49^ were employed for *ab initio* gene model training and prediction, whereas PASA v2.5.2^50^ was used to refine and update gene structures. For homology-based gene prediction, protein sequences of five species from Malpighiales—*Linum lewisii* Pursh (Linaceae),^51^*Kandelia obovata* Sheue & al. (Rhizophoraceae),^16^*Ceriops tagal* (Perr.) C. B. Rob. (Rhizophoraceae),^18^*Passiflora edulis* Sims (Passifloraceae),^17^ and *Chosenia arbutifolia* (Pall.) Skvortsov (Salicaceae)^52^—were used to generate homology gene structures. tRNAs were predicted using tRNAscan-SE v2.0.11.^53^ For functional annotation analyses, motifs and protein domains were determined via InterProScan v5.62-94.0^54^ and EggNOG-mapper v2.1.11^55^ by querying against public databases, including pfam v32.0, gene2product v1.45, interpro v76.0, dbCAN v8.0, busco_outgroups v1.0, merops v12.0, mibig v1.4, go v2023-05-10, repeats v1.0, unipot v2023_02, and eggNOG v5.0. Thereafter, gene prediction and functional annotation results were merged using the annotation module of funannotate. GC content was calculated using bedtools v2.30.0.^56^ Circular plot of chromosomes was visualized using circos v0.69-9.^57^ Hi-C contact map was visualized using HiCExplorer v3.^58^

### 2.8. Phylogenetic analyses, gene family expansion, and contraction

*Averrhoa carambola* L. (Oxalidaceae, Oxalidales)^59^ and *Calophaca sinica* (Fabaceae, Fabales)^60^ were chosen as outgroups in phylogenetic analyses. The longest isoform of each gene from all species was used in subsequent analyses unless stated otherwise. Orthologs among outgroups and five species in Malpighiales were identified using OrthoFinder v2.5.4.^61^ Ortholog sequences were aligned using MAFFT v7.508,^62^ gene trees were inferred using FastTree v2.1,^63^ and species tree with branch lengths was constructed with STAG v1.0.0.^64^ Divergence times were estimated using a penalized-likelihood method implemented in treePL v1.0^65^ based on the species tree. The splitting times of Fabales/Malpighiales, Oxalidales/Malpighiales, and Linaceae/Ochnaceae were set as 110–120 mya, 106–115 mya, and 98–109 mya, respectively, following the dating results of Magallón et al.^66^ Gene family expansions and contractions were inferred using CAFE v5.0.^67^ Gene ontogeny (GO) enrichment analyses were conducted for significantly expanded and contracted gene families using the function ‘enricher’ in the R package clusterProfiler v4.8.2.^68^ All three categories—Biological Process, Molecular Function, and Cellular Component—were included. *P*-values were adjusted using the Benjamini-Hochberg method, and GO terms with an adjusted *P*-value <0.05 were considered significantly enriched.

### 2.9. Genome synteny and population history

Syntenic regions among genomes were identified and visualized using jcvi v1.3.8.^69^ Population size history was inferred using the Pairwise Sequentially Markovian Coalescent (PSMC v0.6.5-r67) model^6^ based on the genome assembly and short-read data of *S*. *rhodoleuca*.

### 2.10. Whole-genome duplication duplicated genes classification

Genome polyploidization events in *S*. *rhodoleuca* and *K. obovata* were inferred using the toolkit WGDI v0.6.5.^70^ Briefly, syntenic blocks were determined based on homologous gene pairs, and synonymous substitution rate (Ks) of these blocks was visualized using the ‘-bk’ option. Gaussian fitting of average Ks values of syntenic blocks was performed with the ‘-kf’ option. Homologous gene pairs derived from different polyploidization events were extracted using WGDI with the ‘-a’ option. These gene pairs were used for GO enrichment analyses via the R package clusterProfiler v4.8.2, as described above. Duplicated gene pairs derived from segmental duplication (SD), tandem duplication (TD), proximal duplication (PD), transposon-derived duplication (TRD), and dispersed duplication (DD) were detected and classified using the R package doubletrouble v1.0.0.^71^*K. obovata* was set as an outgroup in the doubletrouble analysis.

## 3. Results

### 3.1. Genome assembly and annotation

**Figure 1.**
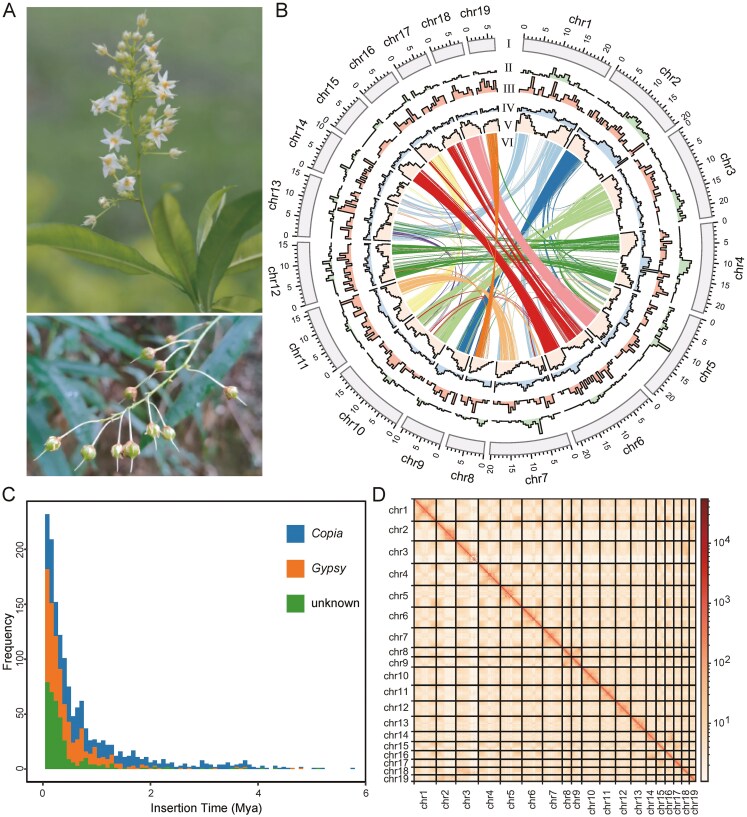
Plant photographs and genome features of *Sauvagesia rhodoleuca*. (A) Inflorescence and young fruits of *S. rhodoleuca*. Photographs were provided by You-Sheng Chen and Xue-Jun Ge. (B) I, 19 pseudo-chromosomes with sequence length ranging from chr19 (6.69 Mb) to chr3 (23.22 Mb); II, *Gypsy* density; III, *Copia* density; IV, GC content; V, gene density; VI, each line indicates a pair of syntenic gene blocks. These metrics were calculated using a window size of 700 kb. (C) LTR insertion time of *S. rhodoleuca*. (D) Hi-C contact heatmap (bin size = 10 kb).

To generate a high-quality *de novo* genome assembly of *S. rhodoleuca*, we employed a combination of ONT long-read sequencing, DNBSEQ short-read sequencing, and Hi-C data. This effort yielded a total of 140.26 Gb of ONT reads, 99.29 Gb of DNBSEQ reads, and 153.99 Gb of Hi-C reads. According to the 21-mer analysis of the DNBSEQ reads, the haploid genome size was estimated to be 243.32 Mb, with heterozygosity of 0.094% and repeat content of 18% (Fig. S1). After quality control of the Nanopore sequencing data, a total of 15,696,077 clean reads and 139.89 Gb of bases were obtained for the genome assembly of *S. rhodoleuca*. The draft genome size was 296.28 Mb, consisting of 53 contigs and a contig N50 length of 15.79 Mb. The redundant sequences in the draft genome were removed, and the remaining sequences were then polished using Nanopore and DNBSEQ reads. Subsequently, clean Hi-C reads (1,026,091,722 reads, 153.74 Gb of bases) were used for scaffolding. After manual adjustment, gap filling, and polish for the scaffolded genome assembly, 99.76% of the sequences with a cumulative length of 290.37 Mb were anchored to 19 pseudo-chromosomes, corresponding to the karyotype of *Saugagesia* (2*n* = 38).^1,72^ The pseudochromosome sizes ranged from 6.69 Mb (chr19) to 23.22 Mb (chr3), with GC content varying from 33.01% (chr17) to 35.46% (chr2) (Fig. 1; Table S1).

The genome completeness was examined by aligning DNBSEQ short reads to the assembly. In total, 91.45% of the short reads were successfully mapped to the genome assembly of *S. rhodoleuca*. According to the BUSCO analysis, 98.40% of the embryophyta core genes were identified as complete (single-copy or duplicated) (Table S2). The LTR assembly index (LAI) was calculated to be 17.28, which falls within the reference-genome quality range (10 ≤ LAI < 20), indicating a high level of assembly completeness and accuracy in LTR retrotransposon representation. The comprehensive quality assessment using CRAQ revealed regional assembly quality indicator (R-AQI) and structural assembly quality indicator (S-AQI) values of 86.84% and 100%, respectively (Table S1), suggesting that our genome assembly achieved high-quality standards (80 < AQI < 90). In addition, the Hi-C contact heatmap displayed clear interaction signals along the diagonal (Fig. 1D), consistent with chromosomal structure, further supporting the accuracy of the assembly and scaffolding.

According to the repeat element annotation, 41.61% (120.83 Mb) of the *S*. *rhodoleuca* genome was identified as repetitive sequences. Within Malpighiales, this proportion is lower than that of *C. arbutifolia* (42.34%),^52^*P. edulis* (86.61%),^17^ and *L. lewisii* (70.5%),^51^ but higher than that of *K. obovata* (24.07%)^16^ and *C. tagal* (35.12%).^18^ Among these elements, LTRs comprised the highest proportion of the genome, including 5.32% of *Copia*, 13.25% of *Gypsy*, and 8.65% of unknown elements (Fig. 1B; Table S3). The insertion of *Copia* and *Gypsy* in *S*. *rhodoleuca* began as early as 5.8 Mya and 4.8 Mya, respectively, with both reaching peak insertion activity around 0.1 Mya (Fig. 1C). This rapid insertion activity led to a substantial accumulation of LTR in the *S*. *rhodoleuca* genome.

Gene prediction was conducted by integrating *de novo*, RNA-seq-based, and homology-based approaches. For RNA-seq-based prediction, we generated 31.52 Gb, 44.09 Gb, and 32.78 Gb of RNA-seq reads from the fruit, leaf, and twig tissues, respectively. For homology-based prediction, we used 36,362 protein sequences from *L. lewisii*, 17,363 from *K. obovata*, 22,203 from *C. tagal*, 38,878 from *P. edulis*, and 32,980 from *C. arbutifolia*. Integrating evidence from all sources, we annotated 26,758 genes, which encode 30,939 proteins. The genomic features revealed an average gene length of 2,900.04 bp and an average CDS length of 230.31 bp. On average, each gene contained 6.43 exons (305.52 bp per exon) and 4.20 introns (307.08 bp per intron). Among the proteins, 28,895 (93.39%) were functionally identified by the EggNOG database, with 26,485 (85.60%), 22,756 (73.55%), and 22,286 (72.03%) of the proteins identified by InterProScan, Pfam, and GO, respectively. In addition to protein-coding genes, 369 tRNA genes were predicted in the genome. According to the BUSCO evaluation, the protein sequences of *S*. *rhodoleuca* had a completeness score of 95.70% (Table S2).

### 3.2. Phylogeny construction and gene family analysis

**Figure 2.**
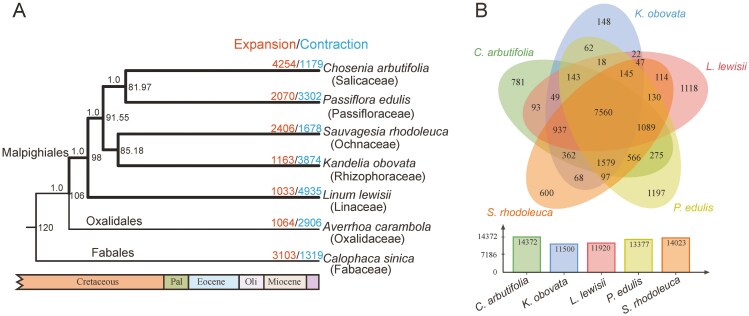
Phylogenetic and gene family analyses. (A) Dated phylogenetic tree of Malpighiales and outgroups. Numbers near nodes indicate divergence times; numbers above internal branches are STAG support values; numbers above terminal branches refer to the count of expanded (red) and contracted (blue) gene families. The colored ribbons below the tree represent the geological time scale. Pal, Paleocene; Oli, Oligocene. (B) The shared and private gene groups among five species in Malpighiales.

A total of 1,235 single-copy orthologs were inferred among *C*. *arbutifolia*, *P. edulis*, *S*. *rhodoleuca*, *K. obovata*, *L. lewisii*, *A. carambola*, and *C. sinica*. All internal branches within Malpighiales received full support (support value = 1.0; Fig. 2A), indicating the robustness of our phylogenetic inference. The species tree showed that *S*. *rhodoleuca* is more closely related to *K. obovata* rather than to *P. edulis* and *C. arbutifolia* (Fig. 2A). According to the dating analysis, Ochnaceae diverged from Rhizophoraceae at the late Cretaceous, 85.18 million years ago (Mya) (Fig. 2A).

Protein sequences of the seven species were clustered and grouped into 19,679 gene families. A total of 14,023 gene families were identified in the *S*. *rhodoleuca* genome, which was slightly less than *C. arbutifolia* (14,372) but more than those characterized in the genomes of *K. obovata* (11,500), *L. lewisii* (11,920), and *P. edulis* (13,377) (Fig. 2B). Moreover, 7,560 gene families were shared by the five species in Malpighiales, whereas there were 600 unique gene families in *S*. *rhodoleuca*, which was greater than that in *K. obovata* (148) but less than that in *C. arbutifolia* (781), *L. lewisii* (1,118), and *P. edulis* (1,197) (Fig. 2B). GO enrichment analysis was performed for the unique gene families in *S*. *rhodoleuca*, which indicated that these gene families were enriched in GO terms ‘terpenoid metabolic process’, ‘isoprenoid metabolic process’, and ‘secondary metabolic process’ (Fig. S2).

According to the gene family analysis, a total of 2,406 and 1,678 gene groups were expanded and contracted in *S*. *rhodoleuca*, respectively (Fig. 2A). Among the expanded gene families, 230 were significant, consisting of 1,419 genes; 192 gene families were significantly contracted with 203 genes. GO enrichment analysis indicated that the significantly expanded genes were enriched mainly in the GO terms ‘monooxygenase activity’, ‘response to oomycetes’, and ‘defense response to oomycetes’, while the significantly contracted genes were enriched in the GO terms ‘plant-type cell wall’, ‘hydrolase activity, hydrolyzing O-glycosyl compounds’, and ‘fatty acid metabolic process’ (Fig. S3).

### 3.3. Genome synteny and historical population dynamics

**Figure 3.**
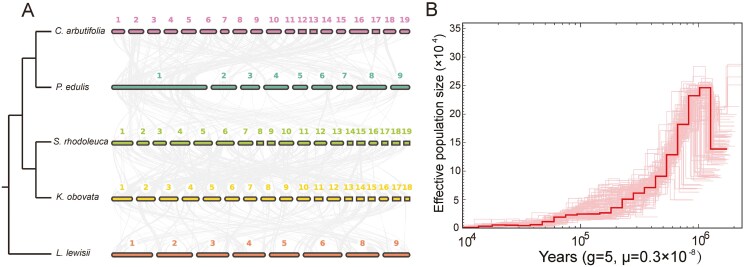
Chromosomal synteny and historical population dynamics. (A) Syntenic plot of five species within Malpighiales. (B) Effective population size of *Sauvagesia rhodoleuca* through time. g, generation time; μ, mutation rate per-site per-generation.

The syntenic plot indicated that substantial chromosomal rearrangements (including inversions, translocations, fusions, or fissions) occurred during the evolution of Malpighiales (Fig. 3A). Historical population dynamic analysis revealed that the effective population size of *S*. *rhodoleuca* peaked around 1 Mya, followed by a rapid decline to the present (Fig. 3B).

### 3.4. Whole-genome duplication events

**Figure 4.**
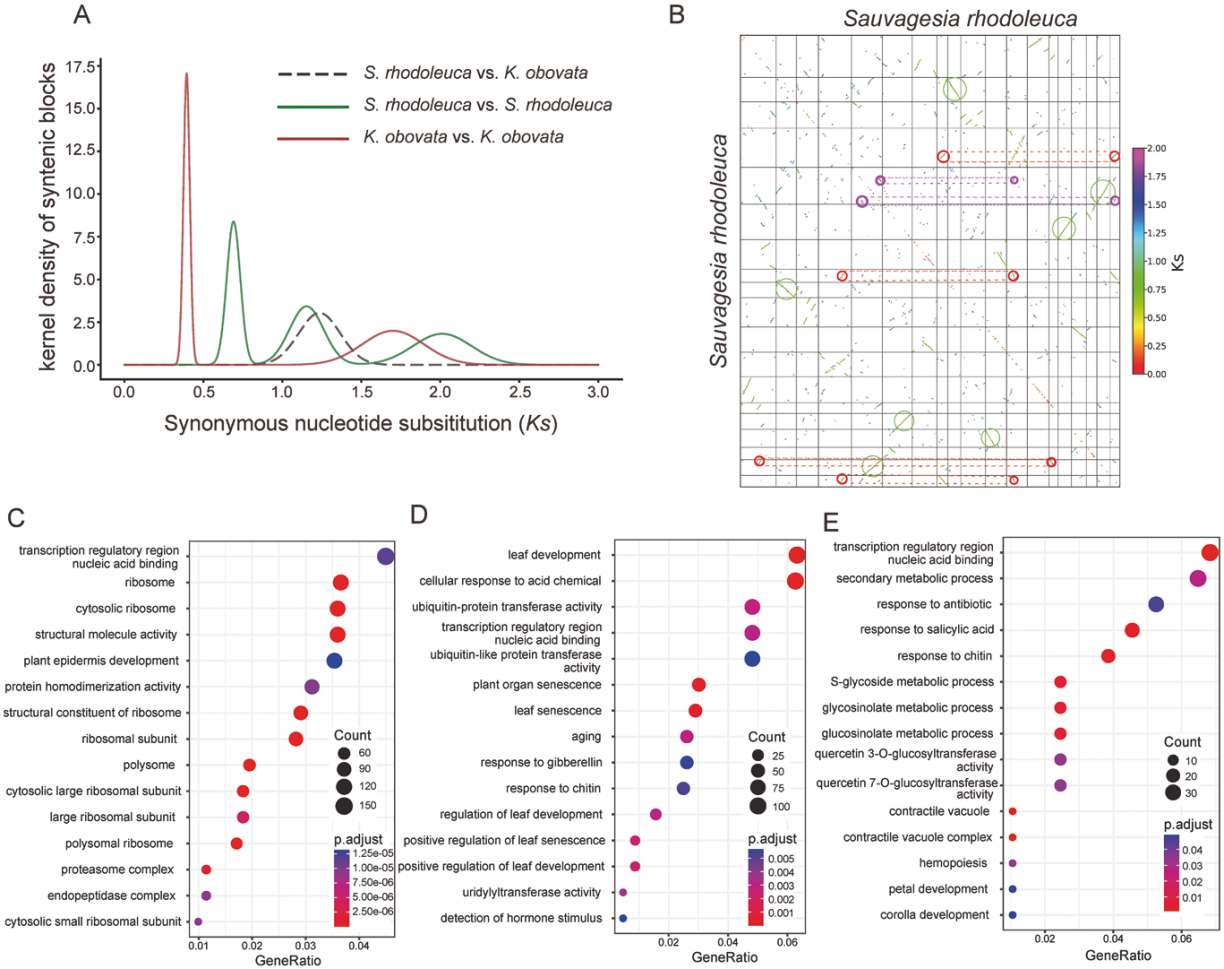
Whole-genome duplication analysis. (A) Distribution of *Ks* between interspecific and intraspecific homologs. (B) Synteny blocks of *S. rhodoleuca*. The axes refer to different chromosomes, the green circle represents the recent WGD event, the red circle represents the WGT event, and the purple circle represents the γ event. (C) GO enrichment of genes derived from the recent WGD. (D) GO enrichment of genes derived from the WGT. (E) GO enrichment of genes derived from the γ event. Count, the number of genes associated with a specific GO term; GeneRatio, the proportion of input genes involved in a specific GO term relative to the total number of input genes.

WGD analysis indicated that *S. rhodoleuca* underwent a recent WGD event and 2 WGT events (Fig. 4A and B). In addition to the *Ks* distribution and dot plot, the inter-chromosomal synteny blocks observed in Fig. 1B are also consistent with a signal of ancient WGDs. Among these, one WGT event, specifically the eudicot-common γ event, was shared by *S. rhodoleuca* and *K. obovata*, whereas the other WGT event occurred shortly after the divergence of *S. rhodoleuca* from *K. obovata* (Fig. 4A). For the *K. obovata* genome, a recent WGD and a WGT (the γ event) were confirmed (Figs 4A and S4). For *S. rhodoleuca*, a total of 5,625, 3,219, and 1,082 genes were derived from the recent WGD, WGT, and the γ event, respectively (Table S4). To investigate the functional roles of these genes in *S. rhodoleuca*, GO enrichment analyses were conducted. Genes derived from the recent WGD event were predominantly enriched in GO terms such as ‘transcription regulatory region nucleic acid binding’, ‘ribosome’, and ‘cytosolic ribosome’ (Fig. 4C). In contrast, genes associated with the WGT event showed enrichment in GO terms including ‘leaf development’, ‘cellular response to acid chemical’, and ‘ubiquitin-protein transferase activity’ (Fig. 4D). Meanwhile, genes originating from the γ event were enriched in GO terms such as ‘transcription regulatory region nucleic acid binding’, ‘secondary metabolic process’, and ‘response to antibiotic’ (Fig. 4E).

Using the ‘full scheme’ mode in doubletrouble analysis, we identified 6,078, 954, 1,562, 18, and 37,503 gene pairs in the *S. rhodoleuca* genome as SD, TD, PD, TRD, and DD, respectively (Table S5). After assigning a unique duplication mode to each gene, 9,133 (SD), 1,350 (TD), 950 (PD), 17 (TRD), and 10,160 (DD) unique genes were categorized (Table S6). GO enrichment analyses were conducted for these unique genes. TD genes were associated with ‘secondary metabolic process’, ‘secondary metabolite biosynthetic process’, and ‘monooxygenase activity’; PD genes with ‘regulation of protein kinase activity’, ‘maturation of SSU-rRNA’, and ‘ribosomal small subunit biogenesis’; TRD genes with ‘regulation of protein kinase activity’, ‘lateral root development’, and ‘regulation of kinase activity’; DD genes with ‘endonuclease activity’, ‘ATP hydrolysis activity’, and ‘catalytic activity, acting on a nucleic acid’ (Fig. S5).

## 4. Disscussion

In this study, we present the first genome assembly of *S. rhodoleuca* in the Ochnaceae family. By integrating Nanopore long-read sequencing and the Hi-C technology, the *S. rhodoleuca* genome was assembled into 19 pseudo-chromosomes, with a total size of 290.37 Mb. Key metrics, including contig N50, mapping rate, BUSCO assessment, LAI, R-AQI, and S-AQI, collectively confirmed the high continuity and completeness of the genome assembly. Furthermore, we inferred species relationships, diversification times, gene family expansion and contraction within Malpighiales, WGD events, as well as the historical population dynamics of *S. rhodoleuca*.

*Sauvagesia rhodoleuca* harbors only 6 populations and exhibits low genetic diversity, pronounced genetic differentiation, and significant isolation among these populations.^4,73^ Challenges in the transition from seeds to seedlings in the wild, combined with limited dry matter accumulation through photosynthesis, lead to exceptionally slow growth rates.^5^ These biological and ecological constraints collectively contribute to its endangered status. This study demonstrated that the effective population size of *S. rhodoleuca* has experienced a continuous decline over the past 1 million years. Its small population size may have accelerated the random loss of genetic diversity, increased vulnerability, and heightened the risk of population decline,^74–76^ as revealed by Chai et al.^77^ and Li et al.^73^ Quaternary climate fluctuations, which significantly influenced vegetation distribution patterns, may have shaped this demographic trajectory. Similar effects were observed in *Cathaya argyrophylla* Chun & Kuang, whose habitat fragmentation and population decline were linked to glacial periods.^78^ These findings underscore the precarious conservation status of *S. rhodoleuca* and provide valuable theoretical insights for future protection efforts.

Numerous studies have demonstrated that the WGD events in angiosperms have played a pivotal role in driving major innovations in morphological traits, stress responses, and even the acceleration of species diversification.^79^ Genes duplicated through WGD events can undergo neofunctionalization, subfunctionalization, back-up compensation, and dosage amplification, contributing to evolutionary adaptability.^80^ In this study, we conducted GO enrichment analyses on genes derived from the recent WGD, WGT, and γ events, respectively. The results indicated that a subset of these genes is involved in critical biological processes, such as ‘plant epidermis development’, ‘leaf development’, and ‘secondary metabolic process’, suggesting that WGD events have contributed to the survival and adaptation of *S. rhodoleuca*.

Phylogenetic relationships within the order Malpighiales have long been a central focus of systematic biologists.^19,21,22^ Despite significant advancements in phylogenetic studies—from analyses based on a few gene regions^15^ to those utilizing hundreds of nuclear genes^22^—the interrelationships among major subclades remain unresolved within Malpighiales. For instance, Xi et al.,^21^ using 82 plastid genes, suggested that Saliaceae and Passifloraceae are more closely related to Linaceae than to Ochnaceae and Rhizophoraceae. Cai et al.,^22^ employing 423 single-copy nuclear genes, proposed that Rhizophoraceae is closer to Salicaceae and Passifloraceae than to Ochnaceae. These conflicting phylogenetic relationships may stem from various biological factors, such as incomplete lineage sorting and hybridization/introgression, or from limitations in the amount of genomic data available.^22,81,82^ In this study, analysis of 1,235 single-copy orthologs revealed that Salicaceae and Passifloraceae are more closely related to Ochnaceae and Rhizophoraceae than to Linaceae, offering novel perspectives on the phylogenetic relationships within Malpighiales. It is important to note, however, that tree topology can be significantly influenced by limited taxon sampling.^83,84^ Given the remarkable diversity within Malpighiales, future studies should prioritize broader sampling at the family level to further refine our understanding of the evolutionary relationships.

## Supplementary Material

dsaf022_suppl_Supplementary_Materials_1

## Data Availability

The ONT reads, Hi-C reads, DNBSEQ short reads, and RNA-seq reads generated in this study have been deposited in the Genome Sequence Archive of National Genomics Data Center, China National Center for Bioinformation under BioProject PRJCA032678 (accession numbers: CRR1386645–CRR1386650) that are publicly accessible at https://ngdc.cncb.ac.cn/gsa, as well as in the Sequence Read Archive of the National Center for Biotechnology Information under BioProject accession PRJNA1273206 (accession numbers: SRR33872230–SRR33872235). The genome assembly, annotations, and coding sequences were deposited in the figshare database (https://doi.org/10.6084/m9.figshare.28794761).
