# Supplementary material for: Chromosome-scale genome assembly of Sauvagesia rhodoleuca (Ochnaceae) provides insights into its genome evolution and demographic history
Source: DNA Res. 2025 Sep 2;32(5):dsaf022. doi: 10.1093/dnares/dsaf022 (PMC12448743; doi:10.1093/dnares/dsaf022)
Supplement: dsaf022_suppl_Supplementary_Materials_1 [file dsaf022_suppl_supplementary_materials_1.zip › Supplemental_table_and_figure_legends.docx]

**Supplemental table legends:**

**Table S1.** Chromosome statistics.

**Table S2.** BUSCO assessment of genome assembly and predicted genes.

**Table S3.** Repetitive elements identified by EDTA.

**Table S4.** Genes derived from the recent WGD, WGT and the γ events.

**Table S5.** Gene pairs derived from SD, TD, PD, TRD, and DD.

**Table S6.** Unique genes derived from SD, TD, PD, TRD, and DD.

**Supplemental figure legends:**

**Figure S1**. Genome survey of *Sauvagesia rhodoleuca*. len, genome length; uniq, unique sequences; het, heterozygosity rate; kcov, k-mer coverage; err, error rate; dup, duplication rate; k, k-mer size.

**Figure S2**. GO enrichment analyses of unique genes of *Sauvagesia* *rhodoleuca*. Count, the number of genes associated with a specific GO term; GeneRatio, the proportion of input genes involved in a specific GO term relative to the total number of input genes.

**Figure S3.** GO enrichment analyses of significantly expanded (A) and contracted (B) gene families. Count, the number of genes associated with a specific GO term; GeneRatio, the proportion of input genes involved in a specific GO term relative to the total number of input genes.

**Figure S4.** Synteny blocks of *Kandelia obovata*. The axes refer to chromosomes.

**Figure S5.** GO enrichment analyses of TD, PD, TRD and DD genes. A) TD genes; B) PD genes; C) TRD genes; D) DD genes. Count, the number of genes associated with a specific GO term; GeneRatio, the proportion of input genes involved in a specific GO term relative to the total number of input genes.
