# Supplementary figures and images for: Chromosome-scale genome assembly of Sauvagesia rhodoleuca (Ochnaceae) provides insights into its genome evolution and demographic history
Source: DNA Res. 2025 Sep 2;32(5):dsaf022. doi: 10.1093/dnares/dsaf022 (PMC12448743; doi:10.1093/dnares/dsaf022)

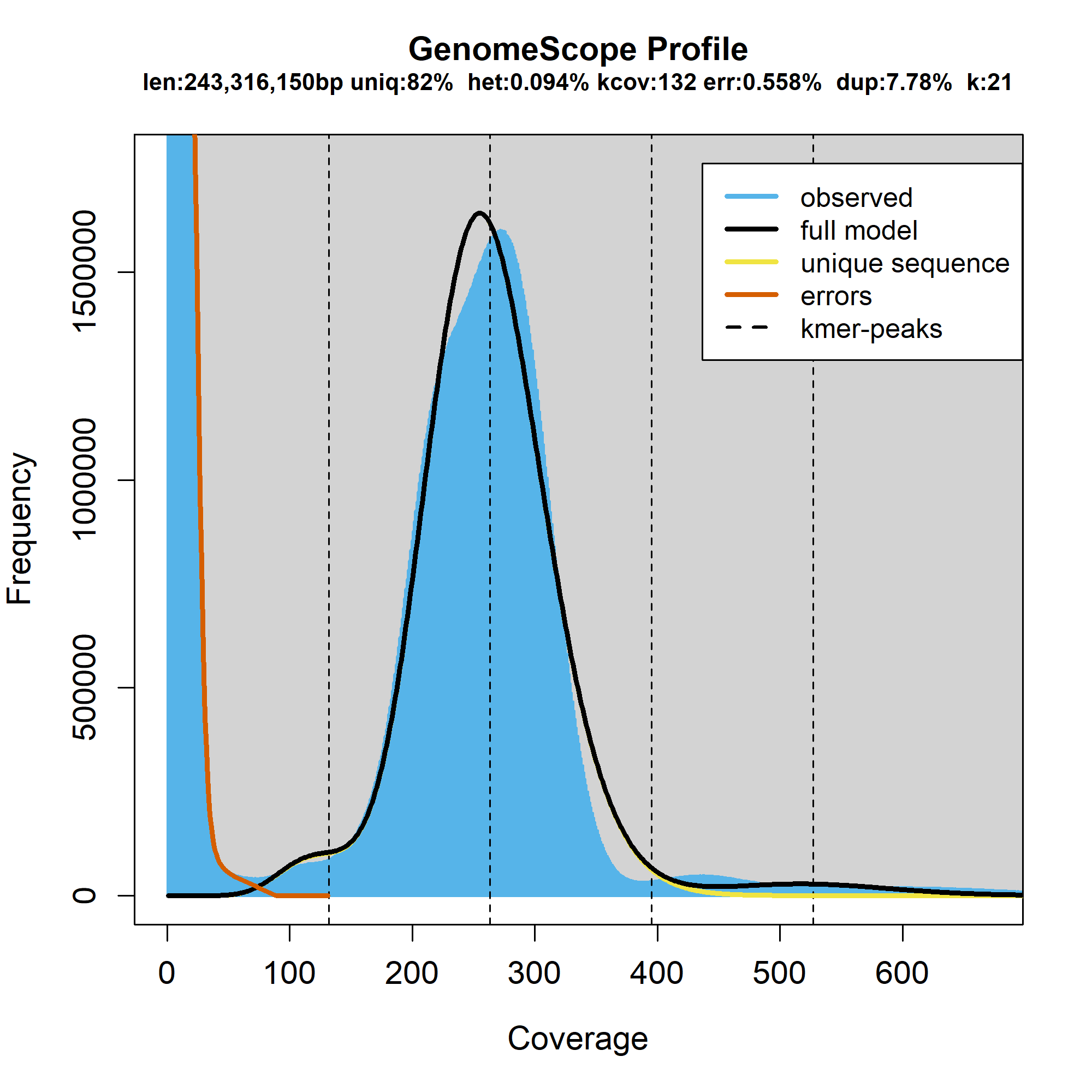

Supplement: dsaf022_suppl_Supplementary_Materials_1 [file dsaf022_suppl_supplementary_materials_1.zip › Figure S1 Genome survey.jpeg]

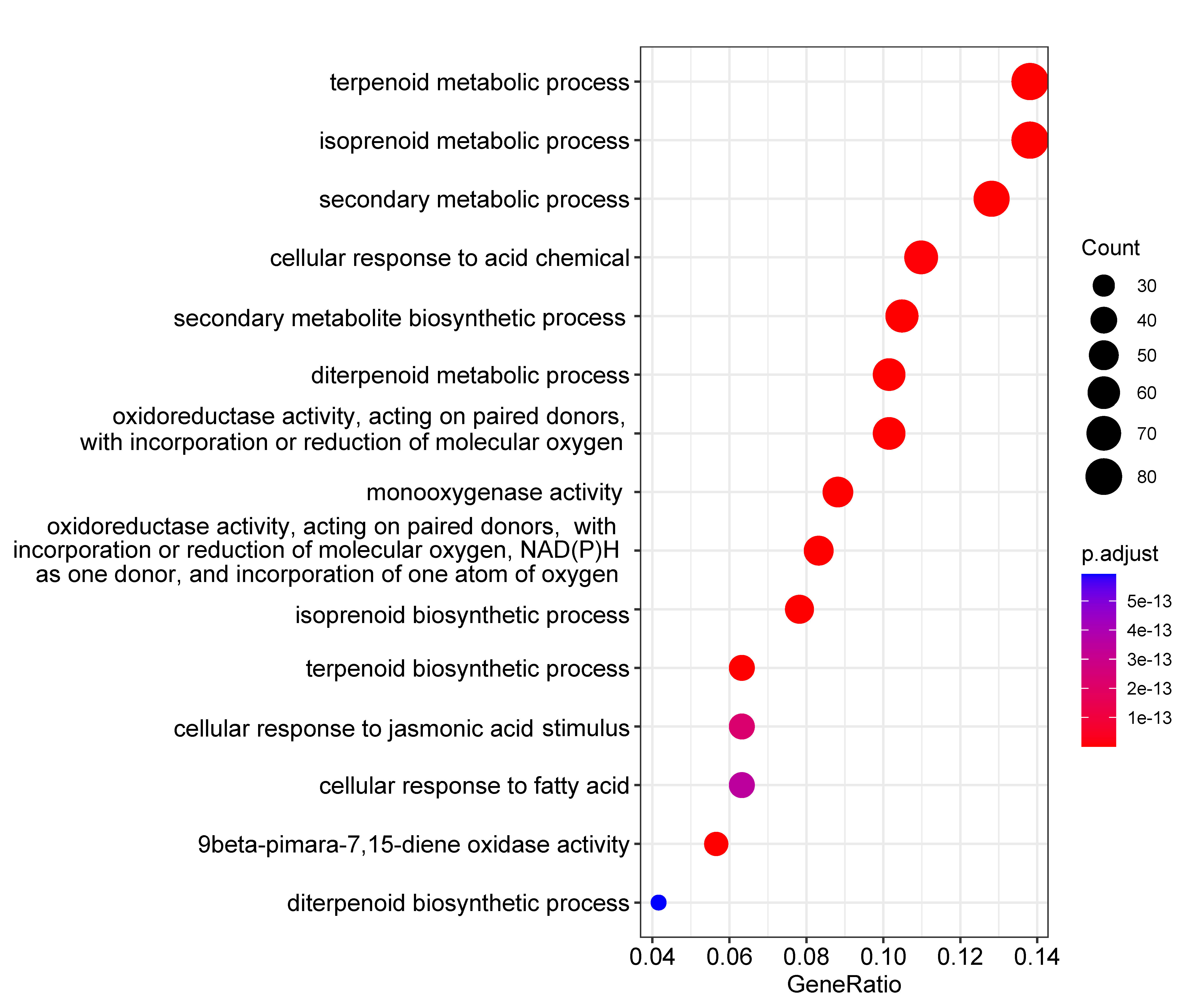

Supplement: dsaf022_suppl_Supplementary_Materials_1 [file dsaf022_suppl_supplementary_materials_1.zip › Figure S2 GO of unique genes.jpg]

A

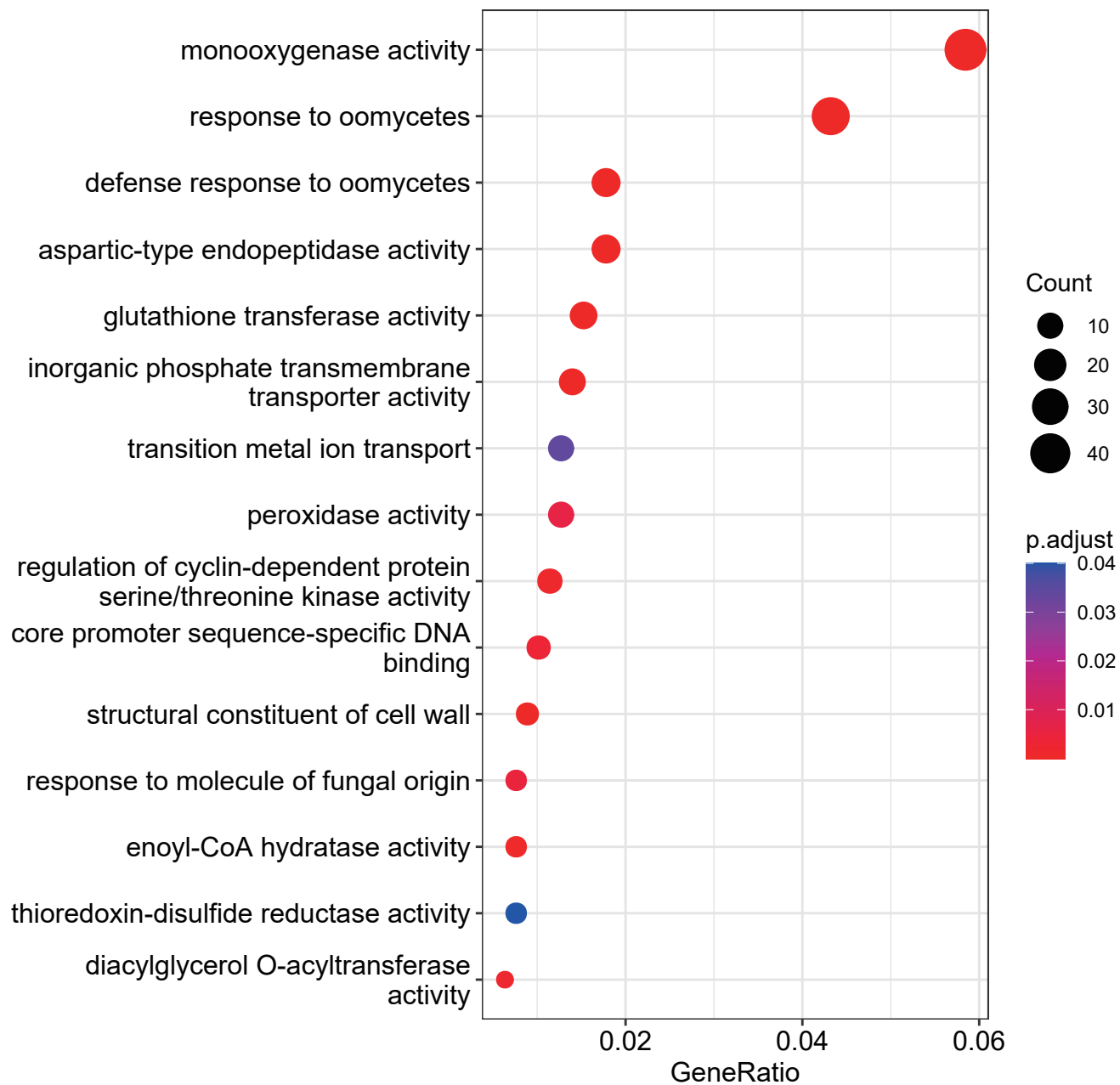

B

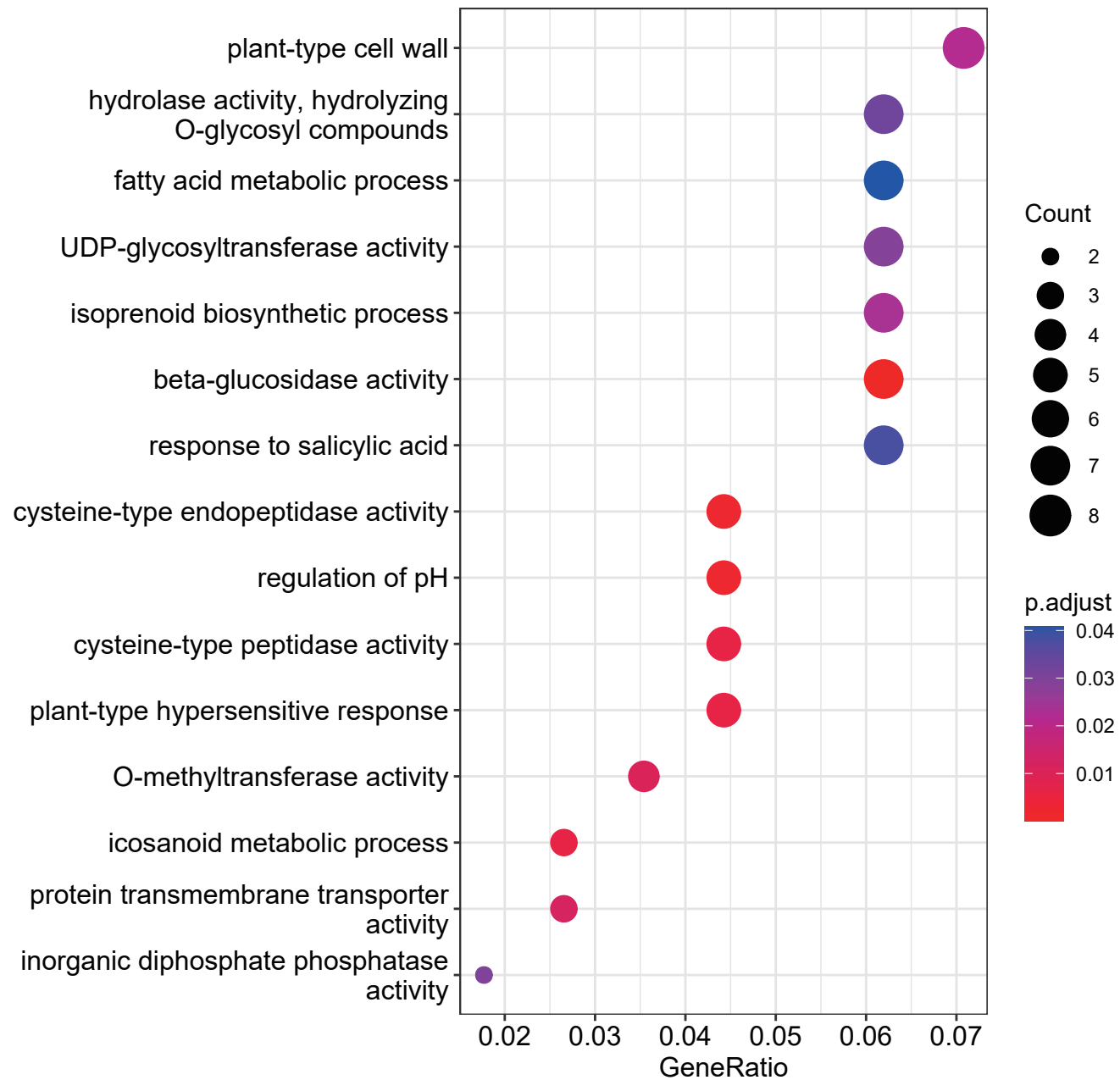

Supplement: dsaf022_suppl_Supplementary_Materials_1 [file dsaf022_suppl_supplementary_materials_1.zip › Figure S3 GO of exp. and contr.pdf]

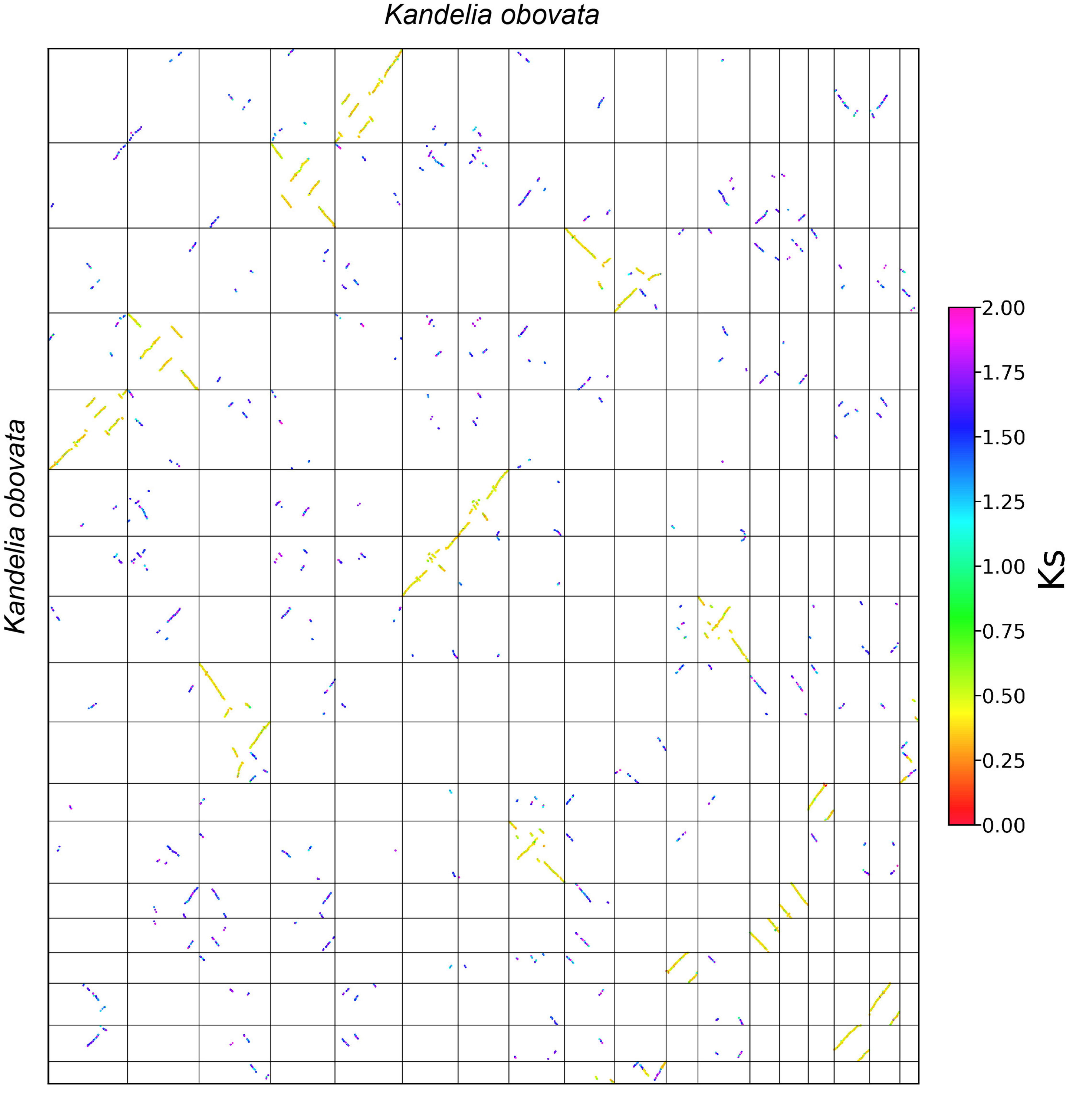

Supplement: dsaf022_suppl_Supplementary_Materials_1 [file dsaf022_suppl_supplementary_materials_1.zip › Figure S4 Kobo.blockks.jpeg]

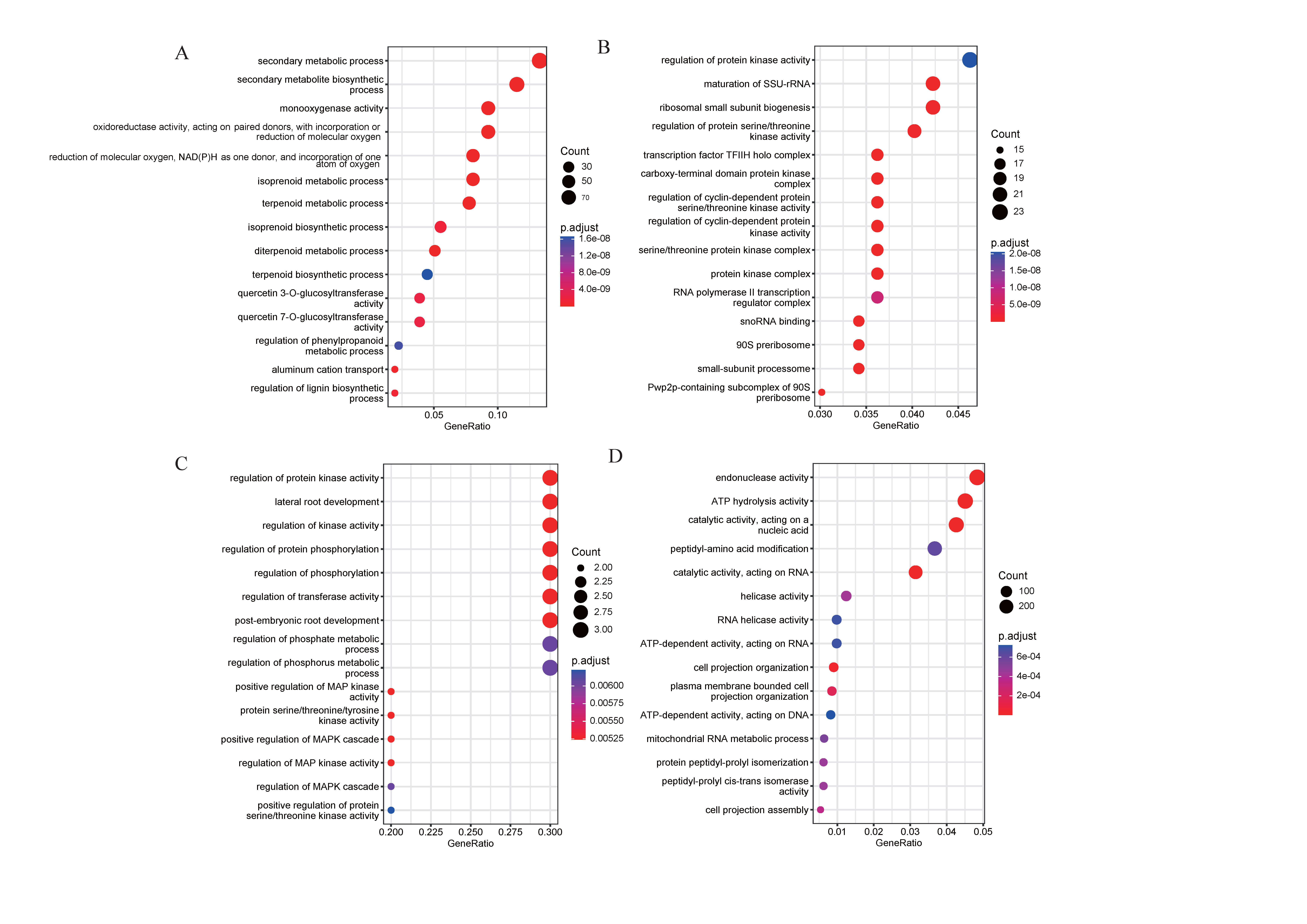

Supplement: dsaf022_suppl_Supplementary_Materials_1 [file dsaf022_suppl_supplementary_materials_1.zip › Figure S5 GO analysis of dup genes.jpg]
